# Sharpening the lens to evaluate interprofessional education and interprofessional collaboration by improving the conceptual framework: a critical discussion

**DOI:** 10.1186/s12909-024-05590-0

**Published:** 2024-06-04

**Authors:** Florian B. Neubauer, Felicitas L. Wagner, Andrea Lörwald, Sören Huwendiek

**Affiliations:** Institute for Medical Education, Department for Assessment and Evaluation, University of Bern, Bern, Switzerland

**Keywords:** Interprofessional education, Interprofessional collaboration, Interprofessional organization, Outcome evaluation, Process evaluation, Modified Kirkpatrick classification, Causal model, Conceptual framework, Terminology, Theory

## Abstract

It has been difficult to demonstrate that interprofessional education (IPE) and interprofessional collaboration (IPC) have positive effects on patient care quality, cost effectiveness of patient care, and healthcare provider satisfaction. Here we propose a detailed explanation for this difficulty based on an adjusted theory about cause and effect in the field of IPE and IPC by asking: 1) What are the critical weaknesses of the causal models predominantly used which link IPE with IPC, and IPE and IPC with final outcomes? 2) What would a more precise causal model look like? 3) Can the proposed novel model help us better understand the challenges of IPE and IPC outcome evaluations? In the format of a critical theoretical discussion, based on a critical appraisal of the literature, we first reason that a monocausal, IPE-biased view on IPC and IPC outcomes does not form a sufficient foundation for proper IPE and IPC outcome evaluations; rather, interprofessional organization (IPO) has to be considered an additional necessary cause for IPC; and factors outside of IPC additional causes for final outcomes. Second, we present an adjusted model representing the “multi-stage multi-causality” of patient, healthcare provider, and system outcomes. Third, we demonstrate the model’s explanatory power by employing it to deduce why misuse of the modified Kirkpatrick classification as a causal model in IPE and IPC outcome evaluations might have led to inconclusive results in the past. We conclude by applying the derived theoretical clarification to formulate recommendations for enhancing future evaluations of IPE, IPO, and IPC. Our main recommendations: 1) Focus should be placed on a comprehensive evaluation of factual IPC as the fundamental metric and 2) A step-by-step approach should be used that separates the outcome evaluation of IPE from that of IPC in the overarching quest for proving the benefits of IPE, IPO and IPC for patients, healthcare providers, and health systems. With this critical discussion we hope to enable more effective evaluations of IPE, IPO and IPC in the future.

## Background

There is scant knowledge on the extent to which the quality of interprofessional education (IPE) and interprofessional collaboration (IPC) at healthcare institutions influences the patient care quality [1–3], the cost effectiveness of patient care, the job satisfaction of healthcare professionals [1] and, as a result, their retention [4, 5]. Patients, people who organize and finance healthcare, policy makers, tax payers, and arguably societies as a whole have a reasonable interest in an answer to this question.

According to the peer-reviewed literature, relevant knowledge gaps persist about the benefits of IPE and IPC despite multiple studies on IPE and IPC outcomes covering a period of almost 50 years [2, 3, 6–13]. Several explanations as to how this can be possible are proposed: The number of evaluation studies is still too low [10]; the time periods typically covered by evaluations is too short to detect final outcomes of IPE/IPC interventions [2, 8, 11, 14, 15]; too much focus is placed on immediate results without including measures for final outcomes from the outset [10]; or, ultimately, positive effects of IPE and IPC simply might not exist [6, 9, 10]. Another frequent and non-contradictory explanation proposes that a lack of clarity in theory and terminology of IPE and IPC and an insufficient use of conceptual frameworks are major deficits which obscure evaluation results [8, 12, 13, 16–21].

In this article, we argue the latter: That an insufficient use of conceptual frameworks has obscured evaluation results. We propose that the persistence of the knowledge gap relating to patient outcomes, satisfaction of healthcare professionals, and cost effectiveness of IPE and IPC activities (briefly, “patient, healthcare provider, and system outcomes”) is rooted in a lack of accuracy in the theoretical models used for mapping causes and effects in IPE and IPC. Our objective is to contribute to overcoming the inconclusiveness in IPE and IPC outcome evaluations by achieving the missing accuracy through the lens of a novel “multi-stage multi-causality” model. Specifically, our research questions are: 1) What are the critical weaknesses of the causal models predominantly used which link IPE with IPC, and IPE and IPC with final outcomes? 2) What would a more precise causal model look like? 3) Can the proposed novel model help us better understand the challenges of IPE and IPC outcome evaluations?

In answering these questions, we first show evidence from the literature that the existing causal models of IPE and IPC exhibit a crucial imprecision. Second, we present the “multi-stage multi-causality model of patient, healthcare provider, and system outcomes” which fixes this imprecision by making a small but important modification to the causal role of IPO. Third, we demonstrate the explanatory power of the multi-stage multi-causality model showing why evaluations using the modified Kirkpatrick classification of interprofessional outcomes (MKC) [11, 22, 23] — a tool commonly used to evaluate outcomes of IPE activities — have failed to substantiate positive outcomes of IPE and IPC; namely, we show how the misuse of MKC leads to inconclusiveness and difficulties in evaluating final patient, healthcare provider, and system outcomes. We conclude with recommendations for future evaluations in the field of IPE, IPO and IPC.

With this theoretical investigation, we hope to contribute to a deeper understanding of the causal factors in IPE, IPO and IPC and to enable more precise evaluations in the future.

## Methods

Based on our research questions, we performed iterative literature searches (detailed below) followed by critical appraisal by the authors, and transformed the resulting insights into the critical discussion presented in the main section of the present article by applying the 6 quality criteria of the SANRA scale [24]:

1. *Justification of the article's importance for the readership:* Our target audience consists of researchers whose goal is to evaluate whether IPE, IPO or IPC improve patient, healthcare provider, and system outcomes. For our target audience the present study is meaningful because it advances the understanding of the theoretical foundations of evaluations in this field. Further, in local contexts where the potential of IPE, IPO, and IPC is still neglected, clear evidence demonstrating substantial benefits would help to foster programs aimed at implementing better IPE, IPO, or IPC.
2. *Statement of concrete/specific aims or formulation of questions*: We set out to explore the following questions: 1) What are the critical weaknesses of the causal models predominantly used which link IPE with IPC, and IPE and IPC with final outcomes? 2) What would a more precise causal model look like? 3) Can the proposed novel model help us better understand the challenges of IPE and IPC outcome evaluations?
3. *Description of literature searches:* We searched for existing definitions, causal models, relevant indicators, and evaluation instruments for IPE, IPO, and IPC using PubMed, Google and Google Scholar with the following search terms in different combinations: “interprofessional education”, “interprofessional collaboration”, “interprofessional organization”, “interprofessional team work”, “evaluation”, “outcome evaluation”, “process evaluation”, “modified Kirkpatrick”, “conceptual framework”, “theory”, “model”, “instrument”, “assessment scale”, “survey”, “review”. We conducted all searches in English, covering the time period from 1950 to 2023. We augmented the initial body of literature found by this strategy with citation tracking: for backward tracking, we followed the references provided in articles which we deemed relevant for our research questions; for forward searches, we used the "cited by" feature of PubMed and Google Scholar. The subchapter-specific literature search used in the development of our definition of IPC is described under “Definition of factual IPC”.
4. *Referencing:* We consistently back key statements by references.
5. *Scientific reasoni*ng: We enable the reader to easily follow our narrative by structuring the present article around the three research questions as stated above, following a logical flow of arguments.
6. *Appropriate presentation of data:* We present the data by distinguishing which findings were taken from the literature and which novel arguments for answering the research questions were derived by us.

### Definitions

#### Definition of IPE

*Occasions when two or more healthcare/social care professions learn with, from and about each other to improve collaboration and the quality of care for patients/clients* [2] (slightly refining the CAIPE definition [25]).

These occasions can happen formally or informally, in dedicated educational settings or at the workplace of healthcare/social care professions, and at any stage along the learning continuum, i.e. foundational education, graduate education, and post-licensure continuing professional development [8, 26]. The central concept in IPE is *learning* [13], the gain of knowledge, skills, and attitudes, or — from a constructivist’s perspective — changes in the brains of individuals.

#### Definition of factual IPC

*Presence of activities in the following 7 dimensions:*

- *Patient-centered care, including a shared treatment plan and effective error management;*
- *Shared creation of the treatment plan and coordination of its execution;*
- *Mutual respect between professions;*
- *Communication, including shared decision-making, sharing of information, appropriate communication tools, and accessibility of team members;*
- *Shared definition and acceptance of roles and responsibilities;*
- *Effective conflict management; and*
- *Leadership, including outcome orientation.*

How did we arrive at this definition? IPC has to be distinguished from traditional “multiprofessional collaboration”. In multiprofessional collaboration, patient care is organized in a discipline-oriented way, affecting its organization, leadership, communication, and decision-making. Different professions work separately, each with their own treatment goals; the physician delegates treatment options to the other healthcare professionals in one-way, mostly bilateral communication [27, 28]. IPC, in contrast, is defined as the occasions “when multiple health workers from different professional backgrounds work together with patients, families, carers and communities to deliver the highest quality of care” [29]. This definition by the WHO remains in use today [30]. However, we found that, in order to talk about specific effects of IPE on IPC and to tailor evaluations towards less ambiguous results, an operationalized definition of IPC is required which provides a higher level of applicability. To create such a definition, we searched the literature to collect a comprehensive list of IPC dimensions which covers all possible settings of IPC. In an iterative process of content-based thematic clustering, reviews, original articles and preexisting questionnaires on the evaluation of IPE and IPC were added until there was agreement between the authors that saturation was reached with regard to all relevant IPC dimensions. This resulted in the following list of publications: [3, 7, 9, 19, 26, 28, 31–39]. Next, we clustered the terms for IPC dimensions found in this body of literature by consensus agreement on sufficient equivalence between three of the authors (FBN, FLW, SH). Clustering was required due to a lack of consistent terminology in the literature and resulted in the comprehensive set of 7 IPC dimensions used in our definition of IPC provided above. Finally, we needed to differentiate IPC from IPE and learning: At the workplace, informal learning happens all the time. As a result, interprofessional work processes can comprise both IPC and IPE at the same time; however, interprofessional learning is only a *possible*, not a *necessary* element of IPC and hence was not included in our definition of IPC. For example, a healthcare professional who is fully equipped with all competencies required for factual IPC could proficiently work in an established team in an interprofessional way without having to learn any additional IPC-related skills.

In order to stress that our definition of IPC includes all the healthcare-related interprofessional work processes actually taking place but excludes the activities required to create them (those fall in the domains of IPE or IPO), we use the term “factual IPC” throughout the present article. Factual IPC not only happens in formal interprofessional work processes like regular, scheduled meetings but also “on the fly”, i.e. during informal and low-threshold communication and collaboration.

#### Definition of IPO

*All activities at a healthcare institution which create, improve, or maintain regular work processes of factual IPC or create, improve, or maintain institutional conditions supporting formal and informal parts of factual IPC, but excluding activities related to IPE.*

There is no agreed upon definition of IPO in the literature, so we propose this refined one here that is broad enough to encompass the full variety of IPC-supporting activities at a healthcare institution while, at the same time, being narrow enough to exclude all manifestations of IPE.

According to this definition, IPO complements IPE within the set of jointly sufficient causes of factual IPC. IPO comprises all conditions required for the realization of factual IPC which are not related to interprofessional learning. It includes the actions of healthcare managers to implement work processes for IPC and to create supportive conditions for IPC (cf. the definitions of IPO in [6, 8, 13, 17, 23, 26, 30, 31, 33, 40–42]). All interventions which establish or improve interprofessional work processes, i.e. which change how things are done in patient care, or which improve the conditions for factual IPC at an institution, belong in the domain of IPO. IPO is also the continued support for factual IPC by management like encouragement, clarification of areas of responsibility, incentives, staffing, room allocation, other resources, or funding. In contrast, established and regular interprofessional tasks themselves, after they have become part of the day-to-day work life of healthcare teams, without requiring further actions by management, would be categorized as factual IPC, not IPO.

Taken together, IPE is the umbrella term for planning, organizing, conducting, being subject to, and the results of interprofessional learning activities, whereas IPO is the umbrella term for all other activities that, in addition to individual competencies of team members, are necessary to cause factual IPC of high quality.

## Critical discussion

### What are the critical weaknesses of the causal models predominantly used which link IPE with IPC, and IPE and IPC with final outcomes?

We start by exploring the models in the literature that describe causes and effects of interprofessional activities in the context of patient care. We will derive evidence from the literature that the existing models exhibit a crucial imprecision regarding the causal role of IPO.

**Fig. 1:**
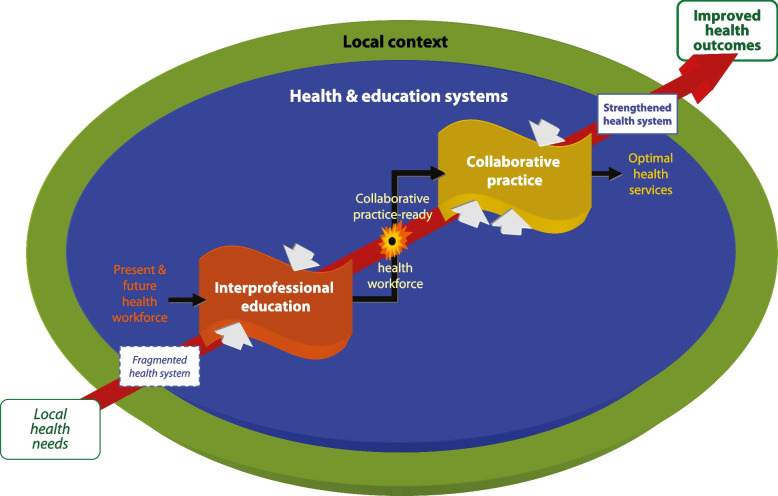
The WHO model of causes and effects in IPE and IPC (from [29], with permission)

The causal model of IPC proposed by the WHO [29, 43] (Fig. 1) was the model predominantly used in past evaluations of IPE and IPC. The WHO model suggests that IPE-related learning leads to IPC competence (knowledge, skills, and attitudes) in the “health workforce” that is “IPC-ready” post-IPE. This readiness “automatically” leads (as the long diagonal arrow in Fig. 1 suggests) to factual IPC. “The World Health Organization and its partners acknowledge that there is sufficient evidence to indicate that effective interprofessional education enables effective collaborative practice” [29]. Factual IPC, in turn “strengthens health systems and improves health outcomes” [29]. As a result, this model suggests a kind of “transitivity” between first causes and last effects: effective IPE activities are expected to ultimately yield positive patient, healthcare provider, and system outcomes on their own.

After its publication, the WHO model was regularly cited and endorsed by IPE experts and continues to exert broad influence today. As of October 6, 2023, the “Google Scholar” search engine showed the original publication [29] to have 4393 citations, 367 of them in 2023 alone.

It is important to note that the WHO model is *monocausal* with respect to IPC, i.e. IPE is the sole necessary cause for factual IPC. While the model acknowledges that, next to IPE, there are further “mechanisms that shape how collaborative practice is introduced and executed”, it only ranks them as supportive: “*Once a collaborative practice-ready health workforce is in place* [emphasis added], these [additional] mechanisms will help them [policy-makers] determine the actions they might take to *support* [emphasis added] collaborative practice” [29]. The following quotes by Reeves and colleagues further illustrate the strong emphasis causal models used to put on IPE: «It is commonly argued that IPE can promote the skills and behaviours required for effective IPC, which in turn can improve quality of health care and patient outcomes” [17] and “National organisations have created core competencies for interprofessional collaborative practice, *positioning IPE as fundamental to practice improvement* [emphasis added]” [10]. A couple of years later, Paradis and colleagues even state: “During this wave [of IPE; 1999–2015], advocates suggested IPE as the solution to nearly every health care problem that arose (…)” [6].

**Fig. 2:**
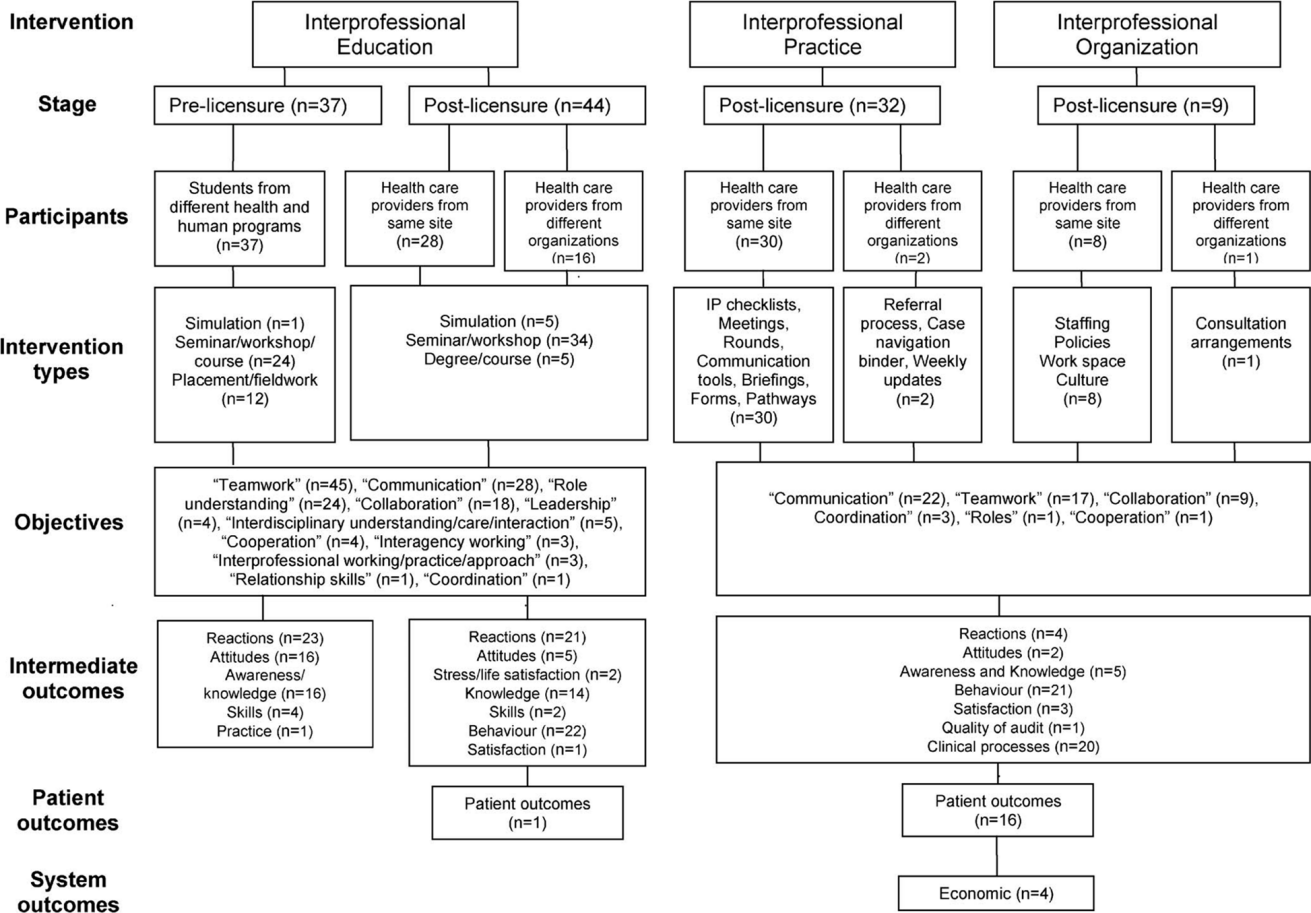
The Interprofessional Framework (from [17], reprinted by permission of the publisher (Taylor & Francis Ltd, [44]). Note that, next to IPE, IPO is listed as a different, additional cause for desired interprofessional objectives and outcomes, but the crucial concept that it also is a *necessary* cause has not yet been worked out here

However, a scoping review by Reeves and colleagues aimed at improving “conceptualization of the interprofessional field” published soon after the WHO model, already acknowledged that the monocausal picture of factual IPC is incomplete [17]. Based on a broad analysis of the literature, their review offers a theoretical “Interprofessional framework” that includes the notion of IPO as an additional and different possible cause for desired interprofessional outcomes (Fig. 2). They define IPO interventions as “changes at the organizational level (e.g. space, staffing, policy) to enhance collaboration and the quality of care”. The explicit inclusion of IPO in this causal model of IPC was a very important step forward. The authors position IPO interventions parallel to IPE interventions, clearly indicating that IPO is an additional *possible* cause for desired interprofessional objectives and outcomes. However, in their framework, the capacity of IPO to be a second *necessary* cause in addition to IPE had not been clearly worked out yet.

Side note: This model and publications using it (e.g. [45]) specify “Interprofessional Practice” (IPP) as a fourth domain, different from IPO (Fig. 2, middle column). However, the IPP elements describe interventions that support work processes of factual IPC, and support for work processes of factual IPC is fully included in our definition of IPO. As a result, we see no necessity to set IPP apart from IPO and do not include IPP as an additional domain in our model below.

For completeness’ sake, we want to mention another explicit model by D’amour and Oandasan [26] with a comparable level of causal clarity which similarly claims that “there are many factors that act as determinants for collaborative practice to be realized”. As this model does not alter our line of argument it is not shown here.

**Fig. 3:**
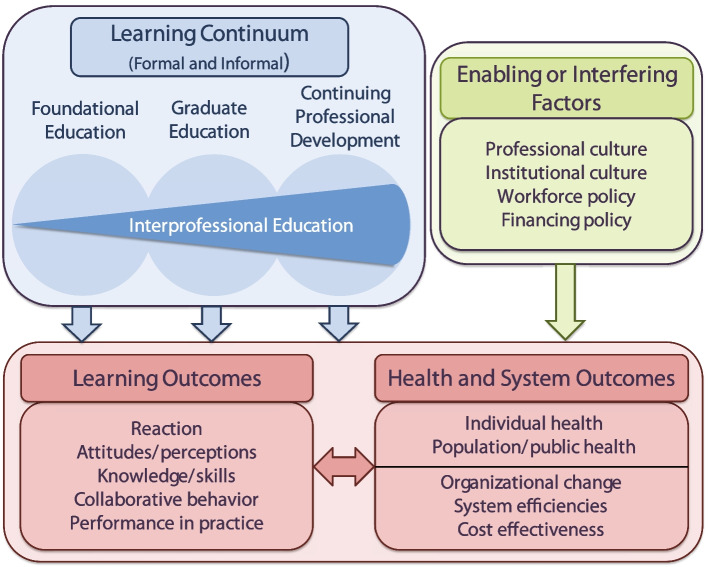
The Interprofessional learning continuum model (from [8], with permission). Under the labels of “Institutional culture”, “Workforce policy”, and “Financing policy” it not only comprises IPO but assigns to IPO the crucial property of being an “enabling” factor, i.e. being co-causal for factual IPC (here labeled as “Collaborative behavior” and “Performance in practice”, lower left row). Despite this important improvement, the hierarchy of causes and effects remains partially vague: **a** The green arrow seems to imply direct effects of IPO on health and system outcomes without acknowledging that if IPO is supposed to have an effect on those at all, it necessarily must improve factual IPC first. **b** The impression remains that factual IPC mainly belongs on the left-hand side, being primarily an effect of IPE. IPO seems less effective on IPC, depending on how one interprets the influence of the green arrow on the larger red box which groups learner, health and system outcomes. **c** The left tip of the red double arrow in the center, indicating an effect of health and system outcomes on learning outcomes, is not discussed in the publication

The ongoing imprecision about the causal role of IPO naturally led to the next iteration of models. The authors of a 2015 review, commissioned by the Institute of Medicine of the National Academy of Sciences (IOM), provide the most recent influential model of causes and effects in IPC which they call “Interprofessional learning continuum model” [8] (Fig. 3).

In comparison to the WHO model (Fig. 1) and the Interprofessional Framework (Fig. 2), this causal model acknowledges that IPO is not just an *additional* but also a *necessary* cause of IPC and thus provides the most elaborate description of the causal relationships between IPE, IPO and IPC in the literature so far. The authors state, “Diverse and often opaque payment structures and differences in professional and organizational cultures generate obstacles to innovative workforce arrangements, thereby impeding interprofessional work. On the other hand, positive changes in workforce and financing policies could *enable* [emphasis added] more effective collaboration (…)” [8]. The word “enable” implies causal *necessity*: if an enabling factor is absent, the effect is disabled, hence the *enabling* factor is *necessary*. The key insight that IPO is a further necessary cause of IPC next to IPE can be found in several other, partly less recent publications, with the only difference that these publications do not embed this insight in a formal model [6, 7, 13, 23, 31, 33, 41, 42]. The causal necessity of IPO becomes evident if one considers the extreme case: imagine a healthcare team whose individual members have all learned through IPE the skill set necessary for high quality IPC, i.e. they are optimally trained for IPC. However, they work at an institution that does not support proper IPC work processes, e.g. there is no dedicated time for team discussions of treatment plans and no electronic tools that allow all team members equal access to patient data. Consequently, there effectively cannot be an optimal manifestation of factual IPC, and it is impossible to expect that the IPE that the team members experienced during their training will significantly affect the quality of patient care in this setting.

### What would a more precise causal model look like?

**Fig. 4:**
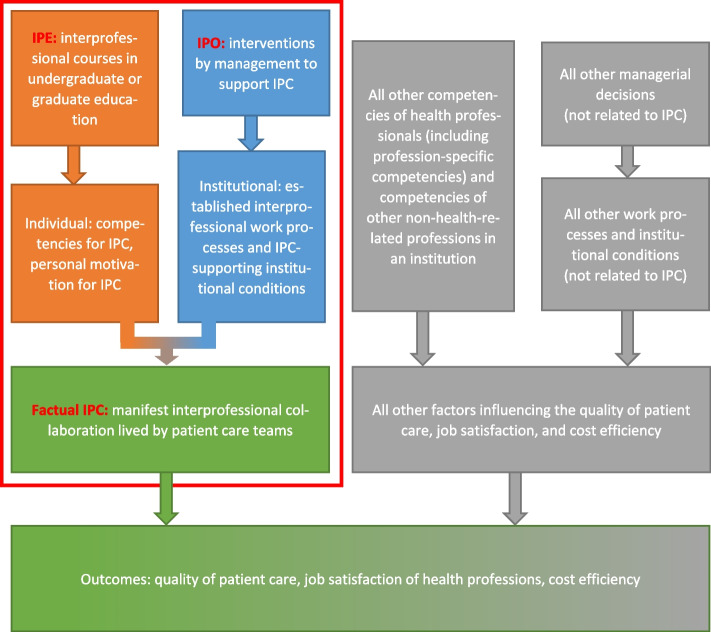
Multi-stage multi-causality model of patient, healthcare provider, and system outcomes. *Key ideas:* IPO is an equally necessary co-factor in the causation of high-quality factual IPC, in addition to IPE. And the entire realm of interprofessional activities (red-outlined box), of which factual IPC is the final and active ingredient, is in turn only one of several causes leading to final outcomes of interest. Orange boxes: Domain of IPE, the domain of acquisition of competencies for IPC by an individual person through learning. Blue boxes: Domain of IPO, defined as the institutional domain of implementation, improvement, and maintenance of work processes of factual IPC and of IPC-supportive institutional conditions. Green box: Domain of factual IPC at a healthcare institution. Green-gray box, bottom row: Final outcomes of interest, i.e. patient care quality, job satisfaction of healthcare professionals, and cost effectiveness of patient care

As we have seen, the notion of IPO in causal models of interprofessionality in the literature progressed from “IPO supportive” (Fig. 1) to “IPO possible but optional” (Fig. 2) to “IPO enabling, i.e. necessary” (Fig. 3). The key result of our study is a refinement missing from the existing causal model of IPE/IPO/IPC. It is the explicit statement that IPO is an *equally necessary* factor next to IPE in the causation of factual IPC. Only jointly are IPE and IPO sufficient to cause factual IPC of high quality. We deem this small modification crucial to reach the conceptual resolution required to fully understand the causes of factual IPC. The fully adjusted causal model is presented in Fig. 4. In this “multi-stage multi-causality model of patient, healthcare provider, and system outcomes”, IPO is now unequivocally labeled as co-necessary for factual IPC alongside IPE-caused individual competencies.

Much more explicitly than previous ones, the multi-stage multi-causality model further shows that there are additional necessary causes for beneficial patient, healthcare provider, and system outcomes *that lie entirely outside of the realm of IPC-related activities* (i.e. outside of IPE/IPO/IPC). It is important to understand that not only factual IPC, but also the final patient, healthcare provider, and system outcomes have more than only one necessary cause, as reflected in the concept of “multi-causality on multiple stages”. This means that optimizing factual IPC is necessary *but still not sufficient* to optimize patient, healthcare provider, and system outcomes. Examples for necessary co-factors on the same level as factual IPC but from outside the realm of IPE/IPO/IPC are a) profession-specific (“uniprofessional”) competencies for aspects of a task that can only be accomplished by members of a specific healthcare profession (*task work* vs. *team work* in [41]), b) details of health insurance policies, which can affect the cost effectiveness of patient care [46], salaries paid to health professionals by a healthcare institution, a factor which can influence job satisfaction [47], or good management decisions at an institution of patient care in general which comprise much more than just full support for factual IPC [46].

It should be noted that the co-causality in this conceptual framework is not compatible with the transitivity of the WHO model, where IPE ultimately leads to patient and healthcare provider outcomes via a predefined chain of “self-sustaining” secondary effects.

In sum, the adjusted causal model proposes that patient, healthcare provider, and system outcomes depend on multi-stage multi-causality. *Stage 1:* IPE + IPO = factual IPC: competencies for IPC in the workforce, the final result of interprofessional learning (IPE), *plus* creating and maintaining IPC work processes and supportive institutional conditions (IPO) *together* cause factual IPC. *Stage 2:* Factual IPC + non-interprofessional factors = patient, healthcare provider, and system outcomes: Factual IPC of high quality *plus* additional necessary but interprofessionality-independent factors *together* cause the final outcomes of interest.

The intention of our notion of “multi-stage multi-causality” is not to devalue the arrow-less “causal halos” of contextual factors in other models but rather to emphasize that even in “complex” systems (systems with multiple interacting elements) the actual sequence of causes and effects should be understood as precisely as possible for optimizing evaluations.

Brandt and colleagues, after reviewing the impact of IPE and IPC, note in their outlook on IPE, “given the complexity of the healthcare world, training learners in effective team work may not ultimately lead to improved health outcomes or reduce the cost of care” [9]. We don’t share this degree of pessimism; above we have shown that a monocausal, IPE-biased view on IPC simply might be insufficient for proper outcome evaluation of IPE and IPC. There is hope that by considering IPO, evaluations will become more conclusive. Wei and colleagues state in a systematic meta-review of systematic reviews about IPC, “Effective IPC is not linear; it does not occur naturally when people come together but takes a whole system’s efforts, including organizations, teams, and individuals” [30]. As we have explained, IPO has to be factored in as an additional necessary cause for IPC, and factors from outside the realm of IPE/IPO/IPC contribute to the “hard” outcomes of interest as well. We presented an adjusted causal model which explicitly acknowledges this multi-stage multi-causality of patient, healthcare provider, and system outcomes.

### Can the proposed novel model help us better understand the challenges of IPE and IPC outcome evaluations?

We claim that the multi-stage multi-causality model exhibits strong explanatory power with regards to the difficulties of showing positive consequences of IPE and IPC in outcome evaluations in the past. To illustrate this, we must first describe the prominent role the modified Kirkpatrick classification of interprofessional outcomes [11, 22, 23] plays in outcome evaluations of IPE and IPC.

#### The modified Kirkpatrick classification (MKC)

MKC is regularly used to classify outcomes of IPE learning activities, curricula and programs [2, 8, 14, 20, 42, 45, 48, 49]. It is a derivative of the original Kirkpatrick model for evaluating training results, named after its author, Donald L. Kirkpatrick, which distinguishes four categories of learning outcomes (Level 1: Reaction, Level 2: Learning, Level 3: Behavior, Level 4: Results) [50, 51]. Expanding the original model, MKC assigns outcomes of IPE activities to six categories [11]:

- Level 1: Reaction
- Level 2a: Modification of perceptions & attitudes
- Level 2b: Acquisition of knowledge & skills
- Level 3: Behavioural change
- Level 4a: Change in organisational practice (wider changes in the organization and delivery of care)
- Level 4b: Benefits to patients/clients

In 2007, the authors of MKC claimed, “We have used these categories since 2000. They have proved useful and, contrary to our initial expectations, sufficient to encompass all outcomes in the hundreds of studies reviewed to date” [11]. This completeness has made MKC a useful tool for authors of review articles as it allows a retrospective classification of IPE outcomes not labeled in the original literature. As a result, MKC was quickly adopted by IPE evaluators around the world to describe the effectiveness of IPE interventions. As Thistlethwaite and colleagues put it in 2015, “This (…) model is now ubiquitous for health professional education evaluation” [42].

At first glance, the existence of such a clear and simple classification of IPE outcomes which not only covers *all* possible IPE outcomes but also is widely embraced in the literature, seems to be good news. What exactly is the problem then? Why did the introduction of MKC more than twenty years ago, plus the conceptual clarification provided by it, not resolve the difficulty in demonstrating IPE-caused patient, healthcare provider, and system outcomes (i.e. effects on MKC levels 4a and 4b)? In the following, we unfold a detailed answer to this question after application of the multi-stage multi-causality model.

#### To achieve progress, IPE and IPC outcome evaluations need to be complemented with process evaluations

MKC classifies outcomes but is agnostic about how these outcomes come into existence. For an evaluator using MKC, the effects of IPE-related interventions unfold inside a black box. The input into the black box is the intervention, the output constitutes 6 different classes of outcomes, i.e. the 6 levels of MKC described above. Naturally, such solely outcome-focused evaluations cannot explain functional interdependencies between the elements of the system. As we have seen, the benefits of IPE and IPC do not unfold as trivially as initially thought. Therefore, after two decades of (overall rather) inconclusive results of applying MKC to the outcomes of interprofessional interventions, the “why” should have moved to the center of the IPE evaluation efforts. This question is posed variously under well-known labels: Authors aware of said stagnancy either call for “formative evaluation” [52], “process evaluation” [14], or “realist evaluation” [42] in order to understand *why* interventions work as intended or not. In the following, we use the term “process evaluation” because we focus on understanding the underlying mechanisms.

#### Process evaluations require a causal model

Process evaluations require a causal model for the system under study to be able to select relevant indicators from a potentially much larger number of conceivable indicators. Appropriately selected indicators, which reflect the inner mechanisms of the system, then replace the black box, reveal bottlenecks, and allow explanations as to *why* interventions did or did not have the expected or intended outcomes. To explicitly demand the use of a causal model in an evaluation is a core principle, for example, of the “realistic evaluation” approach [53]. By directly criticizing the (original) Kirkpatrick model, Holton similarly suggests that a “researchable evaluation model” is needed which should “account for the effects of intervening variables that affect outcomes, and indicate causal relationships” [54]. Specifically for the domain of IPE and IPC, Reeves and colleagues [20] recommend “the use of models which adopt a comprehensive approach to evaluation” and the IOM authors conclude, “Having a comprehensive conceptual model provides a taxonomy and framework for discussion of the evidence linking IPE with learning, health, and system outcomes. *Without such a model, evaluating the impact of IPE on the health of patients and populations and on health system structure and function is difficult and perhaps impossible* [emphasis added]” [8].

#### MKC is not a causal model

Aliger and Yanak note that when Donald Kirkpatrick first proposed his model, he did *not* assert that each level is caused by the previous level [55]. Similarly, the developers of MKC acknowledge that “Kirkpatrick did not see outcomes in these four areas as hierarchical.” Rather, most likely in an attempt to avoid indicating causality in MKC themselves, they talk about “categories” not “levels” throughout the majority of their abovementioned paper [11]. They even knew from the outset that besides IPE the domain which we now call IPO influences outcomes on MKC levels 4a and 4b (but did not include IPO in MKC): “(…) impact of one professional’s changes in behavior depend[s] on [a] number of organisational constraints such as individual’s freedom of action (…) and support for innovation within the organisation” [13]. This means that *by design* neither the original Kirkpatrick model nor MKC are intended to be or to include causal models. MKC simply doesn’t ask *at all* whether additional causes besides an IPE intervention might be required for creating the outcomes it classifies, especially those of levels 3, 4a and 4b. In case such additional causes exist, MKC neither detects nor reflects them. Yardley and Dornan conclude that Kirkpatrick’s levels “are unsuitable for (…) education interventions (…) in which process evaluation is as important as (perhaps even more important than) outcome evaluation” [14].

#### Nevertheless, MKC continues to be misunderstood as implying a causal model

The numbered levels in the original Kirkpatrick model have drawn criticism for implying causality [14, 54, 55]. Originally, Kirkpatrick had used the term “steps” not “levels” [15, 42, 55] whereas all current versions of the Kirkpatrick model, including MKC, now use the term “levels”. Bates [52] cites evidence that Kirkpatrick himself, in his later publications, started to imply causal relationships between the levels of his model. Bates bluntly declares: “Kirkpatrick’s model assumes that the levels of criteria represent a causal chain such that positive reactions lead to greater learning, which produces greater transfer and subsequently more positive organizational results” [52]. Alliger and Janak [55] provide other examples from the secondary literature which explicitly assume direct causal links between the levels and continue to show that this assumption is highly problematic. Most strikingly, the current (2023) version of the Kirkpatrick model [51], created by Donald Kirkpatrick’s successors, *explicitly* contains a causal model which uses the exact same causal logic Alliger and Janak had proposed as underlying it almost 3 decades earlier [55].

As a derivative of the Kirkpatrick model, MKC has inherited just that unfortunate property of implying causality between levels. While starting their above-mentioned publication with the carefully chosen term “categories”, the authors of MKC, in the same publication, later fall back on using “levels” [11]. In earlier publications, they even had *explicitly* assigned explanatory causal power to MKC: “Level 4b: Benefits to patients/clients. This final level covers any improvements in the health and well being of patients/clients *as a direct result* [emphasis added] of an education programme” [22]. Taken together, the authors of MKC themselves, while acknowledging that the original Kirkpatrick model didn’t imply a causal hierarchy, at times contradictorily fuel the notion that MKC provides a viable causal model for the mechanisms of IPE and IPC. As Roland observes, it became common in the literature in general to see the levels of MKC as building on each other, implying a linear causal chain from interprofessional learning to collaborative behavior to patient outcomes [15].

#### Why has the wrong attribution of being a causal model to MKC remained stable for so long?

**Fig. 5:**
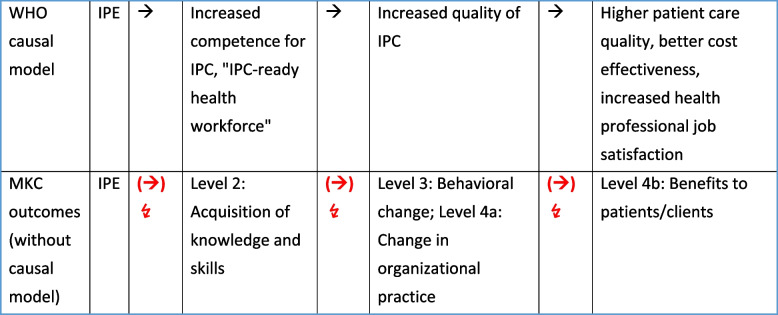
“Unhealthful alliance” between the WHO causal model and MKC. MKC as an outcome classification does not contain a causal model, but uses the term “level” and has numbers attached to each, suggesting causal hierarchy nonetheless. The “levels” of MKC resonate with the causal chain of the WHO model. We speculate that this formal similarity stabilizes the false assignment of a causal structure to MKC (red arrows in the lower row) and, at the same time, as MKC is widely used, perpetuates the use of the WHO model

Why has this misunderstanding of MKC as a causal model not drawn more criticism and why has it been so stable? We speculate that a formal parallelism between the transitive relations in the WHO causal model (Fig. 1) and the numbered levels of MKC, if wrongly understood as a linear chain of subsequent causes and effects, strengthens the erroneous attribution of a causal model to MKC (Fig. 5). Our reasoning: The continued use of the mono-causal WHO model, as opposed to switching to a model incorporating multiple causes for patient, healthcare provider, and system outcomes, stabilizes the misunderstanding of the monothematic (IPE-constricted) MKC as a causal model. (In defense of this mistake, one could say, *if* the transitivity assumption associated with the WHO causal model was true, i.e. *if* the causal chain actually was mono-linear, then MKC *would* be a valid causal model because intermediate outcomes would be the sole causes of subsequent outcomes, covering the entire, linear chain of causes. As a result, there *would* be no difference between outcome evaluation and process evaluation, and MKC *would* be an appropriate tool for process evaluations.) Conversely, we suspect that the wrong but established use of MKC as a conceptual framework in IPE and IPC outcome evaluations stabilizes the continued use of the mono-causal linear WHO model, reinforcing the wrong impression that IPE is the only cause of interprofessional outcomes. The “transitivity” of the WHO model strongly resonates with the observation that the (original) Kirkpatrick model implies the assumption that “all correlations among levels are positive” [55]. If the most upstream event (an IPE activity) is positively correlated with the most downstream elements (patient, healthcare provider, and system outcomes) anyway, why should one bother evaluating intermediate steps? The same fallacy holds true for MKC. When its authors state that “Level 4b (…) covers any improvements in the health and well being of patients/clients as a direct result of an education programme” [22], they not only assign causal explanatory power to MKC, but also neglect the “multi-causality on multiple stages” of outcomes. They assume the same causal transitivity for MKC as is present in the WHO model and thereby expect an “automatic” tertiary effect from an IPE intervention on patient outcomes without considering at all whether the quality of factual IPC – as a necessary intermediate link in the causal chain – has changed due to the intervention or not.

#### If misused as a causal model, MKC does not function and can hinder progress in IPE and IPC evaluations

So far we have established that a) Pure outcome evaluations do not answer the question why it is so hard to detect patient, healthcare provider, and system outcomes of IPE and IPC interventions; b) Process evaluations are required to address this “why” question and to achieve progress in IPE and IPC evaluations; c) A theoretical causal model is required for such process evaluations; d) MKC is *not* such a causal model; e) Nevertheless, MKC falsely keeps being used as such a causal model; and f) The misuse of MKC has remained rather stable, possibly due to a formal parallelism between the WHO causal model and MKC.

The multi-staged multi-causality model of patient, healthcare provider, and system outcomes now makes it clear why evaluations which implicitly or explicitly treat MKC as a causal model are bound to fail in their *process* evaluation part: MKC, when used as a causal model, is crucially incomplete: In terms of the causes of factual IPC (cf. Figure 4, orange and blue boxes), MKC sees IPE but is blind to IPO; and in terms of the direct causes of patient, healthcare provider, and system outcomes (cf. Figure 4, green and grey boxes), MKC sees factual IPC but is blind to the complementary non-interprofessional causes because none of its levels covers them. MKC is a classification limited to detecting outcomes of IPE, and neither IPO nor non-interprofessional factors are such outcomes. When speaking about the original model (but with his statement being transferable to MKC), Bates notes that “Kirkpatrick’s model implicitly assumes that examination of (…) [contextual] factors is not essential for effective evaluation” [52]. Citing Goldstein and Ford [56], he continues, “when measurement is restricted to (…) the four (…) levels no formative data about why training was or was not effective is generated” [52]. Specifically targeting the MKC version, Thistlethwaite and colleagues imply that MKC lacks IPO: “When thinking of applying of Kirkpatrick’s framework to IPE, we must remember the importance of the clinical environment (…) and consider how conducive it is to, and facilitative of, any potential change in behaviour arising from interprofessional learning activities” [42].

Bordage calls conceptual frameworks “lenses” through which scientists see the subjects of their studies [57]. Following this metaphor, we conclude that the resolution of the “conceptual lens” of MKC, if misused as a causal model, is too low for process evaluations. In our perspective, this, in turn, is the most likely reason why outcome evaluations of the past have failed to reliably demonstrate terminal benefits of IPE and IPC.

It is important to note that MKC by design solely, agnostically and successfully measures outcomes of interprofessional education in different dimensions. Therefore, its failure to detect bottlenecks in IPE and IPC is not its own fault, but the fault of evaluators who continue to use it as a causal model while failing to acknowledge the multi-staged multi-causality of patient, healthcare provider, and system outcomes.

We next take a closer look at how exactly MKC fails. In the mono-linear, low-resolution view of MKC, if a study that evaluates the effects of an intervention fails to detect final outcomes, the only logical possible conclusion is to question the effectiveness of previous levels. If there are changes in interprofessional behavior (level 3) but there is no benefit to patients (level 4b), the conclusion is that changes in interprofessional behavior are not beneficial to patients; if there are interprofessional competencies acquired by learners (level 2) but no subsequent change in interprofessional behavior (level 3), then interprofessional competencies do not translate into behavior. Using MKC as the conceptual lens, the logical answer to “why” is that “the training program was not designed in ways that fostered effective transfer or (…) other input factors blocked skill application” [52], and a straightforward overall conclusion with regards to the knowledge gap about the benefits of IPE and IPC would be that IPE is not very effective in terms of patient, healthcare provider, and system outcomes. While this disappointing result has actually been considered as a possibility [6, 9, 10], more often alternative explanations are sought in an attempt to rescue IPE efforts and to avoid the conclusion that IPE is ineffective while sticking with MKC as the causal model.

One of these “escape routes” is to claim that it is methodologically too difficult to measure outcomes on MKC levels 3, 4a and 4b by using different variants of a temporal argument. Paraphrasing Belfield et al. [58], Roland [15] states that “patient outcomes may only become apparent over a *protracted period of time due to the time needed for the learner to acquire and implement new skills* [emphasis added by us, also in the following quotations]” whereas Hammick and colleagues state, “It is unsurprising that all but one of the studies (…) evaluated IPE for undergraduate students. The *time gap* between their interprofessional learning and qualification clearly presents a challenges [sic] associated with evaluating levels 3, 4a and 4b outcomes” [11]. Yardley and Dornan add, “early workplace experience (…) *might take months or even years* to have any demonstrable effect on learners, let alone patients” [14]. The IOM comments that “Efforts to generate this evidence are further hindered by the *relatively long lag time* between education interventions and patient, population, and system outcomes” [8] while Reeves and colleagues note that “*the time gap* between undergraduates receiving their IPE and them qualifying as practitioners presents challenges with reporting outcomes at Levels 3, 4a, and 4b” [2]. The core argument here is always that undergraduate IPE happens in educational institutions whereas IPC happens at the workplace at healthcare institutions *much later*. By this logic, the causal chain assumed by MKC might be fully intact but the time lag between an IPE intervention and effects on levels 3, 4a and 4b constitutes an insurmountable methodological difficulty and renders comprehensive evaluations of IPE outcomes impossible.

Another “escape route” is to invoke “complexity” of IPE as the reason why its final outcomes are hard to detect. Thistlethwaite and colleagues [42] agree with Yardley and Dornan [14] that the MKC is not suited to evaluate “the *complexity* of health profession education and practice.” The authors from the IOM state that “The lack of a well-defined relationship between IPE and patient and population health and health care delivery system outcomes is due in part to the *complexity* of the learning and practice environments” [8]. The term “complexity” usually refers to systems which are cognitively difficult to understand because they have many elements or because science has not figured out yet how to model their interactions [59]. In our opinion, the term “complexity” in the context of IPE is ill-defined and a placeholder for saying that the set of causes of patient, healthcare provider, and system outcomes is not being understood well and that a more precise causal model is required to figure out what is going on.

#### Compare and contrast: “multi-stage multi-causality” as causal model

If we use “multi-stage multi-causality” as the conceptual lens instead of MKC we increase the available resolution and can see more elements of the system. If evaluations fail to show beneficial outcomes of IPE or IPC, we now can do much better asking the right sub-questions to find an answer to “why”. Viewed through the high-resolution lens of the multi-stage multi-causality model, the list of possible failure points on this trajectory significantly expands. The resulting high-resolution picture provides an exquisite set of novel testable hypotheses (Table 1). Collecting data on different levels, including the level of factual IPC, should enable decisions as to which of these scenarios were attributable to an IPE intervention having no multi-level effect.

**Table 1:** Novel testable hypotheses, based on the multi-stage multi-causality model, if interprofessional outcome evaluations are inconclusive

a)	The particular IPE intervention was inappropriate to cause learning; no subsequent effects in the causal chain are possible
b)	The particular IPE intervention was effective in causing learning; however, contrary to the assumption, interprofessional learning does not improve factual IPC; no subsequent effects possible (in this case it does not matter whether supporting IPO was present or not)
c)	The IPE intervention was effective in causing learning; however, the lack of IPO (no work processes or favorable work conditions were created or improved) denied the effect of IPE on factual IPC and prevented improvement in factual IPC; no subsequent effects possible
d)	The IPE intervention was effective in causing learning; supporting IPO was present; factual IPC improved; however, contrary to the assumption, factual IPC does not improve the final outcome under study, e.g. cost effectiveness
e)	The IPE intervention was effective in causing learning; supporting IPO was present; factual IPC improved. Nevertheless, improved factual IPC was not able to improve patient outcomes because the lack of necessary co-factors like uniprofessional skills of team members was the actual bottleneck and blocked improvement of the final outcome under study, e.g. patient health

A made-up example for case c): An interprofessional team attends a commercial workshop and learns how to communicate details of the treatment plan using a modern patient information system. Learning at this workshop constitutes the IPE intervention. Back in the hospital, the patient information system is implemented but, due to cost considerations (IPO decision), only the “light” version is bought, in which a crucial element of document sharing is missing. As a result, the team members cannot use the software in the effective way they learned to. In the end, the IPE intervention fails to have an effect on patient outcomes because factual IPC was not improved due to the lack of a co-necessary IPO activity, i.e. buying the “full” software version

Taken together, we argue that the answers to “why” allowed by the low resolution lens of MKC when misused as a causal model might sometimes be wrong and should be replaced with more detailed explanations.

1. It is premature to conclude that IPE has no effects on patient, healthcare provider, and system outcomes unless the presence or absence of all co-causes has been considered.
2. The deeper cause of the temporal argument might be to mistakenly use MKC as a causal model because the use of MKC masks any problems with IPO or other co-causes. Given the higher resolution of the multi-stage multi-causality model, it is now possible to conceptually distinguish between the known challenge arising from the passage of time (creating various confounders) and the case in which a lack of IPO blocks the effects of IPE. It should be possible, in principle, to assess at any later point in time, for example by means of a survey, how much and which types of IPE members of an interprofessional team had experienced earlier in their career and how much they remember; or even to assess their current competencies for IPC in a practical exam. Such measurements might reveal that individual competencies for IPC are present, no matter how much time has passed since their acquisition, and that IPO is the actual bottleneck.
3. Likewise, alleged methodological perplexity due to IPE “complexity” is de-emphasized if we swap the low-resolution lens of MKC for the high-resolution lens of the multi-staged multi-causality model. The high-resolution picture (Fig. 4; Table 1) replaces the fuzzy placeholder of “complexity” by adding missing elements of the system to the model.

In sum we have demonstrated that when MKC is misused as a causal model it neglects co-causing factors with essential influence on IPE outcomes, is therefore an insufficient tool to detect bottlenecks, and edges out any better-suited, viable causal model. This miscast hampers meaningful process evaluations, the subsequent improvement of indicators and interventions, and thereby ultimately the progress in proving beneficial patient, healthcare provider, and system outcomes of IPE and IPC.

### Limitations

One limitation of our theoretical critical discussion is that we did not illuminate how hard it is to quantify patient, healthcare provider, and system outcomes from a methodological point of view (e.g. document-based patient data analysis). Neither did we address the extent to which this limits the meaningfulness of IPE/IPC outcome evaluations. However, we claim that the conceptual weakness of missing co-causalities is the deeper root of the evaluation problem, not particular methods, and that methodological issues are solvable as soon as relevant co-causalities are appropriately considered.

Another limitation is that a model is always a simplification. For example, the multi-stage multi-causality model does not include personality traits of team members, intra-personal abilities like self-regulation, or the harmony of personality types within a team, which also play a role in factual IPC. These traits would be difficult to incorporate into the model and gathering such information for evaluations might even be unethical. Similarly, the model does not reflect the influence which the behavior and health literacy of patients (and their families, caregivers, and communities) might have on factual IPC.

A third limitation is that we did not discuss a particular setting in which the use of MKC as mono-linear causal model could work, namely, if IPE champions themselves become IPO managers and subsequently establish factual IPC in their institutions through an appropriate combination of IPE and IPO. In this scenario, the roles of health professionals (as carriers of IPE-induced competence for factual IPC) and managers (as IPO decision makers) overlap – obviously potentially optimal to foster factual IPC. In a certain sense, in this particular case, IPE would lead to IPO and to factual IPC with the potential of “transitively” improving patient, healthcare provider, and system outcomes. However, as we believe that there is no fixed relationship between undergoing IPE and becoming a healthcare manager, we did not pursue this line of argument further, regarding it as an exception.

## Conclusions

In our critical discussion we have analyzed previous models of causes and effects in IPC based on the existing literature, proposed a novel “multi-stage multi-causality” model, and demonstrated its explanatory power by establishing that MKC is not suited to foster progress in proving or disproving beneficial final outcomes of IPE and IPC. We conclude with 6 practical, applicable recommendations for future IPE, IPO, and IPC outcome evaluations.

### Recommendation 1: stop (mis-)using MKC as a causal model

We have pointed out that the continued use of MKC as causal model seems to severely inhibit the scientific exploration of the co-necessity of IPO and non-interprofessional factors and therefore delays answering the important question whether IPE and IPC *actually* improve patient, healthcare provider, and system outcomes. As early as 1989, the use of the original Kirkpatrick model as a causal model was questioned [55]. In 2004, Bates took the position that the continued use of this model is unethical if beneficial results are missed by evaluations due to the narrow focus on outcomes [52]. Today, we conclude that using MKC as a causal model in IPE, IPO or IPC outcome evaluations should be discontinued.

### Recommendation 2: state the causal model under which evaluations of IPE/IPO/IPC operate

Evaluators should make an explicit statement about the causal model under which they design interventions and interpret results, including their additional assumptions about the chain of causes and effects. Knowledge of these assumptions allows the reader to detect inconsistencies – an important element for causal clarification – and should prevent the field of IPE, IPO, and IPC outcome evaluations from getting mired down for even more decades.

### Recommendation 3: always include some process evaluation

Even if the primary goal of a study is summative outcome evaluation, evaluators should always include some process evaluation to test the causal model they assume and under which they designed their evaluation, and do so at least until the topic of causality in IPE, IPO, and IPC is fully settled. For example, if an IPE intervention aims at improving factual IPC, evaluators who assume multi-causality would co-evaluate IPO to make sure that IPO is no bottleneck in the evaluated setting.

### Recommendation 4: strive for specificity in IPE, IPO, or IPC interventions

If the only goal of an intervention is to improve a certain outcome metric like patient safety, one might initiate a broad, non-specific intervention using best-practice guidelines and all available resources. However, if a goal of the intervention is also to show the existence of specific benefits of IPE, IPO, or IPC in a scientific way, then the multi-causality of outcomes must be taken into account. Intervention designs that change both, interprofessional and non-interprofessional causes of outcomes, must be avoided. For example, if uniprofessional training (a cause outside the domain of IPE/IPO/IPC) is also part of an intervention (e.g. the re-design of the entire workflow in an emergency department in order to enhance patient safety), then this mix of causes obscures the contribution of IPE, IPO, or IPC to the desired effect. Reeves and colleagues euphemistically and aptly call measuring the particular influence of IPE on patient outcomes in such multifaceted interventions a “challenge” [10]. This example shows why theoretical clarity about the causal model is required to effectively evaluate beneficial outcomes of IPE, IPO, or IPC. Respecting the multi-stage multi-causality of patient, healthcare provider, and system outcomes means designing interventions that improve interprofessional elements only, or, if other components inevitably change as well, to control for those components through comprehensive measurements and/or by adding qualitative methods that allow final outcomes to be causally attributed to IPE, IPO, or IPC.

### Recommendation 5: always quantify factual IPC

Recommendations 5 and 6 are our most important recommendations. It is self-explanatory that without the emergence of factual IPC there cannot be any final, globally desirable outcomes of upstream IPE or IPO activities; not until IPE or IPO activities improve factual IPC, does the attempt to evaluate their effects on patient, healthcare provider, and system outcomes start to make any sense. Further, *if* a positive correlation exists between the quality of factual IPC and patient, healthcare provider, and system outcomes, then correlating factual IPC with final outcomes is the most conclusive way to show it. While the notion that factual IPC is the minimum necessary condition for final outcomes of interprofessional efforts is not new [8, 19], the realization that the attached transitivity assumption (that IPE automatically creates the necessary IPC) is wrong, certainly is. As shown above, dismissing transitivity is a cogent consequence of embracing the multi-stage multi-causality of final outcomes. In future evaluations, the quantification of IPE therefore should no longer serve as a surrogate for the quantification of factual IPC. Rather, factual IPC, as an intermediate necessary step towards final outcomes and their most direct cause within the realm of IPE/IPO/IPC, always needs to be evaluated on its own. The same holds true for future evaluations of IPO. IPO interventions do *not* automatically lead to factual IPC, but first must be shown to improve factual IPC before they can be expected to cause any changes in patient, healthcare provider, and system outcomes. Taken together, a comprehensive measurement of the quality of factual IPC needs to be the centerpiece of any meaningful evaluation of final outcomes achieved by IPE interventions, IPO interventions, combined IPE + IPO interventions, or of factual IPC itself.

From the large number of dimensions of factual IPC (see “Methods”) arises the necessity to evaluate it in detail. Such completeness in the evaluation of factual IPC is important for several reasons:

- *Obtaining a meaningful sum score:* Evaluating factual IPC in a given setting against a hypothetical optimum requires integration of *all* of its subdimensions into one sum score.
- *Not missing correlations:* If an IPC score does not cover all dimensions of factual IPC, correlations between factual IPC and its effects (or causes) might be missed, even if these relationships truly exist. *Example:* An evaluation which only includes the dimensions of “mutual respect” and “conflict management” might miss an actually existing correlation between factual IPC and cost effectiveness, mainly driven, say by the dimension of “shared creation of the treatment plan and coordination of its execution”. The result of this evaluation could cast substantial doubt on the existence of positive effects of factual IPC despite them actually being there. Similarly, only a complete set of IPC indicators is suited to reveal potentially *diverging* effects of different subdimensions of factual IPC on different final outcomes. For example, optimal interprofessional team behavior that maximizes patient safety, might, at the same time, turn out to be *less* cost effective than multiprofessional team behavior that compromises on patient safety.
- *Optimizing process evaluation:* A complete IPC coverage further provides valuable information for process evaluations aimed at identifying weaknesses in factual IPC. Significant correlations between outcomes and specific subdimensions of IPC can suggest causal relationships and uncover crucial components for successful IPC in a given setting. Focusing on strengthening these subdimensions could help optimize patient, healthcare provider, and system outcomes.
- *Enabling setting independence and comparisons:* Factual IPC is setting-specific [11, 19, 35, 60, 61], i.e. the needs of patients for specific medical services differ across different contexts of patient care (e.g. emergency care; acute care; rehabilitation; chronic care; multimorbid patients; palliative care). As a consequence, different subdimensions of factual IPC contribute to the outcomes of interest to a variable degree depending on the specific healthcare setting. Even *within* a specific setting, requirements and behaviors necessary for effective IPC can vary due to the specifics of the case, e.g. the particular rareness or severity of the patient’s condition. Assumptions made prior to an evaluation about which subdimensions of factual IPC are most important in a specific setting therefore should not preclude the exploratory evaluation of the other subdimensions. If an evaluation grid misses IPC subdimensions, it may work well in one setting but fail in others. Hence, the completeness of indicators for factual IPC in an evaluation instrument creates setting independence, eliminates the burden of adjusting the included IPC subdimensions every time a new healthcare setting is evaluated, and allows unchanged evaluation instruments to be re-used in subsequent studies (called for by e.g. [16]) as well as multi-center studies (called for by e.g. [2]). A starting point for the operationalization of factual IPC including all of its subdimensions is provided in our definition of factual IPC (see “Methods”; a validated evaluation toolbox based on this operationalization will be published elsewhere; a published tool which also covers all subdomains of factual IPC, with a focus on adaptive leadership, is the AITCS [39]).

### Recommendation 6: use a step-by-step approach for proving benefits of IPE and IPO

The multi-stage multi-causality of patient, healthcare provider, and system outcomes naturally implies that the process of proving that IPE or IPO benefits final outcomes could be broken down into discrete steps. The key idea is to evaluate the impact of interprofessional activities on each of the subsequent levels in the causal chain while controlling for non-interprofessional factors. Showing the effects of IPE on IPC competencies, the effects of IPC competencies on factual IPC, and the effects of factual IPC on patient, healthcare provider, and system outcomes then becomes *three different research agendas* that can be processed independently. If it can be shown in the first of these research agendas that IPE leads to learning (by controlling for non-interprofessional learning-related factors), and in the second, independent research agenda, that learning leads to improved factual IPC (controlling for IPO), and in the third research agenda that factual IPC leads to desired final outcomes (controlling for co-conditions for final outcomes like uniprofessional competencies), then the benefit of IPE on patient, healthcare provider, and system outcomes is ultimately proved. If this approach fails, then at least it will be exactly revealed where the chain of effects breaks down. The same holds true for IPO: Show that IPO interventions lead to work processes and/or favorable institutional conditions which support factual IPC, separately show that these work processes and conditions lead to improved factual IPC (if co-conditions for factual IPC like IPE are present), and show that better factual IPC leads to an improvement of final outcomes; then the positive impact of IPO is verified.

By covering the entire process, this “step-by-step” approach could build a compelling case for how interprofessional interventions lead to desired final outcomes. It further could markedly simplify the agenda of interprofessional research because it takes the burden of showing the effect of one particular IPE or IPO intervention on one particular final outcome off the shoulders of evaluators. After breaking down the evaluation task into separate steps that prove the impact from link to link, researchers are free to work on *one* step at a time only.

#### Outlook

The presented critical discussion advances the theoretical foundations of evaluations in the field of IPE, IPO and IPC. To improve patient-centered care by means of IPC, one needs to think bigger than just training of healthcare professionals in the competencies and mindsets required for effective IPC; work processes also have to be established and optimized in a setting-dependent manner to allow for factual IPC to happen. Besides IPC, factors like discipline-specific knowledge of health professionals or administrative aspects of patient management have to be optimized, too, to achieve optimal patient, healthcare provider, and system outcomes.

By sharing the multi-stage multi-causality model and its pertinent theoretical clarification we hope to contribute to a deeper understanding of causes and effects in interprofessional collaboration, to answer the repeated call in the research community for improved theory in this field, to explain difficulties faced by past evaluations, and to provide helpful guidance for future research studies. Our key recommendations for future evaluations of interprofessional outcomes are to focus on a comprehensive evaluation of factual IPC as the most fundamental metric and to deploy a step-by-step research agenda with the overarching goal of proving beneficial patient, healthcare provider, and system outcomes related to IPE, IPO, and IPC. With these contributions, we hope to help healthcare institutions improve their evaluations of IPE, IPO, and IPC, ultimately benefiting health, healthcare provider, and system outcomes.

## Data Availability

No datasets were generated or analysed during the current study.

## Abbreviations

IPC: Interprofessional collaboration
IPE: Interprofessional education
IPO: Interprofessional organization
MKC: Modified Kirkpatrick classification
WHO: World Health Organization
